# Incidence of asymptomatic COVID-19 positivity in cancer patients and effects on therapy

**DOI:** 10.21203/rs.3.rs-1785577/v1

**Published:** 2022-07-01

**Authors:** Lisa Liu, Nicole M Ross, Elizabeth A. Handorf, Caitlin R. Meeker, Giana Chen, Donald Baldwin, Namrata Vijayvergia

**Affiliations:** Fox Chase Cancer Center

**Keywords:** asymptomatic COVID-19, COVID-19 screening, intervention delay, treatment delay, pandemic

## Abstract

**Purpose:**

The coronavirus disease 2019 (COVID-19) pandemic is posing unprecedented challenges for patient care, especially for cancer patients. This study looks at asymptomatic (AS) COVID-19 positivity in cancer patients and its effects on their care.

**Methods:**

We conducted a retrospective chart review of AS patients testing positive for COVID-19 upon screening at Fox Chase Cancer Center between January 2020 and September 2020. Relationships between positive tests and demographics, clinical characteristics, and treatment delays were investigated using conditional logistic regression or Mantel-Haenszel tests.

**Results:**

Among 4143 AS patients who underwent COVID-19 testing, 25 (0.6%) were COVID-19 positive (cases) and these were matched to 50 controls. The median age was lower in the cases compared to that of the controls (64 vs 70 years old, p = 0.04). Of the cases, 10 patients (40%) never underwent their planned oncologic intervention [6/10 (60%) did not require the planned intervention once deemed okay to proceed]. Of the controls, only 1 patient (2%) did not undergo the planned intervention. Of these 15 COVID-19 positive patients who underwent the planned intervention, 11 (73.3%) had a delay related to COVID-19, with a mean delay duration of 18 days (range: 0–49, SD: 16.72).

**Conclusion:**

Cancer patients had lower incidence of AS COVID-19 than general population. Delays that occur due to AS COVID screening are not very long and serve as a tool to limit spread of virus. Further studies will be important in addressing delays in cancer care and concerns of patient safety as the pandemic continues.

## Introduction

Severe acute respiratory syndrome coronavirus-2 (SARS-CoV-2) is the causative agent of our current global pandemic. SARS-CoV-2 causes COVID-19, typically affects the upper and lower respiratory tract, and can lead to severe pneumonia(Muralidar et al. 2020). Individuals who are older or have prior comorbidities are particularly susceptible to severe disease(Muralidar et al. 2020). Due to human-to-human transmission, authorities encourage individuals to social distance, self-isolate, and quarantine, and health care facilities to suspend most non-urgent medical procedures in an effort to slow down the spread of this virus(Shah, Nogueras, et al. 2020). With such measures, COVID-19 has posed unprecedented challenges on patient care, especially for patients with complex illnesses, such as cancer. This is not only because oncology care delivery increases opportunities for viral transmission, but also because delay of such care, though not life-threatening immediately, can lead to catastrophic downstream effects(Kutikov et al. 2020). While patients need to receive their cancer treatments, going to clinic poses an increased risk of catching and/or spreading COVID-19. To make matters worse, cancer patients have unique challenges in this pandemic, due to immunosuppression from treatments, surgeries, or the disease itself(Liang et al. 2020). Accumulating evidence suggests that cancer patients have an increased risk for SARS-CoV-2 infection and increased morbidity and mortality(Al-Shamsi, Coomes, and Alrawi 2020). The presentation of COVID-19 and its disease course is unpredictable as new variants appear(Torjesen 2021).

One of the major challenges of controlling the COVID-19 outbreak is the transmission of COVID-19 from patients with AS COVID-19(Wang et al. 2020). The incidence of AS COVID-19 cases in the general population was estimated to be 17%(Byambasuren et al. 2020). The clinical characteristic of individuals with AS COVID-19 is having no clinical symptoms or chest imaging findings^(Gao et al. 2021)^. To identify AS COVID-19, universal screening measures including SARS-CoV-2 polymerase-chain reaction (CovPCR) testing prior to invasive interventions and therapy administration were instituted to address risk of spread from AS COVID-19 patients across many cancer centers in the United States. If patients tested positive for COVID-19, they were instructed to quarantine for 14 days and monitor symptoms while their cancer directed therapy was postponed. There is limited data published on the incidence and outcomes of AS COVID-19 in cancer patients and the resulting delay in cancer care for patients. The purpose of this study protocol is to perform a retrospective chart review to study the demographics (age, sex, race, ethnicity, employment status), clinical characteristics (cancer diagnosis, presence of comorbidities, current symptoms) and effects on oncologic care of AS patients who tested positive for COVID-19 upon screening.

## Methods

### Study subjects

In this retrospective study, AS cancer patients who underwent CovPCR testing between March 2020 and September 2020 were included. This analysis compared cases (positive CoVPCR) identified by screening of AS patients to controls (negative CoVPCR). Each positive COVID-19 screen was randomly matched to 2 negative controls, using exact matching (without replacement) on month of test, cancer type and planned intervention type. AS cancer patients were defined as patients with cancer who had no self-reported symptoms of fever (≥ 100.5°F), cough, headache, loss of taste/smell, shortness of breath, diarrhea, or high-risk exposure. The primary objective of this study was to determine the incidence of AS COVID-19 in cancer patients treated at Fox Chase Cancer Center and its implications. The study was approved by the Institutional Review Board.

### Data Collection

Patient demographics and clinical characteristics were extracted through chart review. Demographic information includes age at date of COVID-19 testing, sex, race (white, black, other, unknown), Hispanic/Latino ethnicity, and employment status. Clinical characteristics include type of cancer diagnosis and presence of comorbidities including cardiovascular disease, connective tissue disease, neurologic disease (including cerebrovascular accidents), and venous thromboembolism. For the cases, chart review was conducted to assess if they were symptomatic around the time of testing (presence of fever, fatigue, dyspnea, sore throat, congestion, runny nose, nausea, vomiting, and diarrhea), had a recent hospital/ER visit for COVID related issues, and if they became symptomatic within 14 days of testing.

Data evaluating the effect of AS COVID-19 on planned interventions (chemotherapy, endoscopic procedures, surgery, and radiation therapy) was also collected and quantified to define “delay” (actual date of intervention - planned date of intervention). We compared the delay in receiving the intervention in AS COVID-19 patients with matched controls (patients who tested negative for COVID-19). Finally, we evaluated the viral load in positive COVID-19 cancer patients and its relationship to symptomatology in the following 2 weeks.

### Statistical Methods

Patient characteristics were summarized with descriptive statistics and frequency distributions. The associations between positive COVID-19 screen and age, gender, race, diagnosis, cardiovascular disease, connective tissue disease, venous thromboembolism, symptomatic, fever, dyspnea, and diarrhea were tested with conditional logistic regression or Mantel-Haenszel tests (stratified by matched set). Mantel-Haenszel tests were also used to determine the association between positive COVID-19 screen and intervention occurrence and intervention delay. Statistical tests were not performed on variables with substantial missingness or little variation (i.e. ≥95% in most common category). Analyses were completed using R version 3.6 (R Foundation for Statistical Computing). Statistical significance was defined as a 2-sided P-value < 0.05.

## Results

Between March 2020 and September 2020, 4143 AS patients underwent CoVPCR testing. Only 25 AS cancer patients tested positive for COVID-19, an incidence of 0.6%. 75 patients were used for analysis: 25 cases; 50 controls (Table 1). The median age was lower in the cases (64 vs 70 years old, p = 0.04). Sex and race were similar between cases and controls. The predominant cancer types in the cases and controls combined were genitourinary (33.3%), gastrointestinal (20%) and breast (13.3%). Cancer type, comorbidity distribution, and symptoms were similar between cases and controls. Of the cases, 10 patients (40%) never underwent the planned oncologic intervention while 15 patients (60%) did. Of these 10 patients, 3 patients suffered cancer related mortality and one had infectious complications. The remaining patients no longer required the specific procedure (kyphoplasty, endoscopic procedure, and skin biopsy) after resolution of COVID-19 infection. In the control arm, only 1 patient (2%) did not undergo the planned intervention.

The 15 patients that did undergo the planned oncologic intervention were then matched to 30 controls (Table 2). Of these 15 patients, 11 (73.3%) had a delay related to the positive CoVPCR (2 patients had no intervention planned, 2 patients had the intervention performed the same day). The mean duration of delay was 18 days (range 0–49 days, SD 16.72) in cases versus zero days in control.

Of the 25 cancer patients who were CoVPCR positive, 19 patients (76.0%) had no symptoms (PCR mean: 93.0 range: 83.5–106.7), two (8.0%) had symptoms at the time of CoVPCR testing (PCR mean: 82.3 range: 79.9–84.7), and four (16.0%) had symptoms emerge within 14 days of CoVPCR testing (PCR mean: 98.0 range: 88.7–112.0). For these three groups, the quantitative PCR values (cycle threshold) did not predict whether or not the patient would have symptoms (p-value = 0.133).

## Discussion

From this study, we found an incidence of AS COVID-19 patients among patients with cancer at our academic cancer center in Pennsylvania, USA to be 0.6%. Only a few others have addressed this important issue. For instance, a study done in Dubai, United Arab Emirates found that the incidence of AS COVID-19 in patients with cancer was 8% (7 of 85 patients)(Al-Shamsi, Coomes, and Alrawi 2020), while a study from New York, United States found an incidence of 0.74% (4 out of 537 patients)(Shah, Mayer, et al. 2020). Our rate of AS COVID-19 in cancer patients was similar to that found in the study by Shah et al performed in New York, United States. These rates of AS COVID-19 in cancer patients are much lower compared to that in the general population, in a meta-analysis paper found the estimated incidence to be 17%^(Byambasuren et al. 2020)^. Patients with cancer tend to already take measures to try to prevent infection, including physical distancing, avoiding crowds, and avoiding anyone who is sick. It is likely that rates of COVID-19 in cancer patients are less than that in the general population because of such increased protective health measures. Although lower than that of the general population, AS COVID-19 in cancer patients still needs to be caught early on to prevent further spread.

Our study found that 40% of the COVID-19 positive patients did not undergo their planned intervention and others had a delay in their care but the median duration of this delay was less than 3 weeks (18 days, with a range of 0 to 49 days). The institution of pre-procedure screening for COVID-19 may lead to inevitable procedural delays but the degree of delay may be acceptable for a wider public health benefit of widespread screening and protecting our vulnerable patients. Many studies have looked at how COVID-19 pandemic has affected cancer care delivery due to overwhelmed medical systems diverting their priorities. For example, in a global survey with 1,051 respondents from 84 countries looking at the effects of the COVID-19 pandemic on the management of colorectal cancer patients, 745 (71%) respondents reported a delay in care. Of these, 48.9% had change in initial surgical plan, 40.3% refused surgery during the COVID-19 emergency phase, and 26.3% had originally planned for elective operations but then needed urgent surgery^(Santoro et al. 2021)^. Prior studies have shown that colorectal cancer surgery can be safely delayed beyond the normal wait time up to 4 weeks without having a significant impact on patient survival or cancer progression^(Turaga and Girotra 2020)^. Santoro et al’s paper, it was noted that 58.3% of respondents reported that COVID-19 prolonged the diagnosis-to-treatment interval to ≥ 5 weeks beyond the normal wait time^(Santoro et al. 2021)^. A recent study assessing the impact of COVID-19 on cancer related deaths estimated that there may be a 17% increase in colorectal cancer specific death rate during this time in the United Kingdom^(Maringe et al. 2020)^. Instituting measures to prevent spread of the virus will ensure timely care for all cancer patients and screening for AS COVID is one such step.

Widespread testing has been part of the strategy to improve the safety of treating of cancer patients during the pandemic. Specifically, at our academic institution, we instituted screening for all staff and patients (temperature checks and query of symptoms), limited the number of visitors, and spaced out our infusion rooms and waiting room areas. If a patient needed an urgent intervention despite testing positive, the staff was able to plan, minimize contact with other patients, bring the patient in at the end of the day and then deeply clean the room. Patients who did not require an in-person visit were offered a tele health visit with their doctors.

The institution of vaccination programs has significantly reduced the risk to patients and providers alike but with new variants emerging and waning immunity from prior vaccinations, reinstitution of such screening measures may be required in the future. Our and other studies like this provide data to support such screening procedures in the setting of high transmission rates and emerging new variants.

The findings of this study should be interpreted in light of some study limitations. First, this study was a single-center retrospective review and may not be representative of the cancer patients at other cancer centers across the United States. Second, we were limited by a relatively small sample size of AS COVID-19 positive cancer patients so a larger sample size may be helpful to more clearly elucidate some of the trends that we see. Nevertheless, this study shows novel findings and trends among cancer patients who have AS COVID-19 and how this affected their care.

## Conclusion

Incidence of AS COVID-19 in our cancer patients was significantly lower than that of the general population. Active screening delayed oncologic care marginally but the institution of such measures and other precautions (separate treatment rooms and scheduling procedures at the end of the day for infected patients) have ensured safe and prompt cancer care delivery during the pandemic. With these measures and patient education, we maintained the outpatient clinical arena largely free of SARS-CoV-2. Further studies will be important in addressing the widespread impact of such practices on patient care delivery and safety as the pandemic continues.

## Figures and Tables

**Table 1:** Demographic and clinical characteristics of Cases (positive screening for COVID-19) and Controls (negative screening for COVID-19). NS = non-significant, NC = not calculated. Statistical tests were not performed on variables with substantial missingness or little variation (i.e. ≥95% in most common category)

Characteristic	Controls (%) (n = 50)	Cases (%) (n = 25)	Total (%) (n = 75)	p-value
Age, mean (range)	70.1 (46.0–93.0)	64.1 (35.0–84.0)	68.1 (35.0–93.0)	0.04
Male	24 (48%)	7 (28%)	31 (41.3%)	NS
Race				
Black/African American	5 (10.0%)	5 (20.0%)	10 (13.3%)	NS
White	41 (82.0%)	15 (60.0%)	56 (74.7%)	NS
Other	1 (2.0%)	2 (8.0%)	3 (4.0%)	NS
Unknown	3 (6.0%)	3 (12.0%)	6 (8.0%)	NS
Hispanic/Latino	0 (0.0%)	1 (4.0%)	1 (1.3%)	NC
Employed for wages	16 (32.0%)	6 (24.0%)	22 (29.3%)	NC
Cancer type				
Gastrointestinal	10 (20%)	5 (20%)	15 (20%)	NS
Genitourinary	19 (38%)	6 (24%)	25 (33.3%)	NS
Lung	6 (12%)	0 (0%)	6 (8%)	NS
Breast	7 (14%)	3 (12%)	10 (13.3%)	NS
Gynecologic	1 (2.0%)	8 (32.0%)	9 (12.0%)	0.046
Head/Neck	2 (4.0%)	2 (8.0%)	4 (5.3%)	NS
Other	5 (10.0%)	1 (4.0%)	6 (8.0%)	NS
Cardiovascular disease	28 (56.0%)	16 (64.0%)	44 (58.7%)	NS
Connective tissue Disease	4 (8.0%)	0 (0.0%)	4 (5.4%)	NS
Neurologic problems	0 (0.0%)	1 (4.0%)	1 (1.3%)	NC
Venous thromboembolism	1 (2.0%)	2 (8.0%)	3 (4.0%)	NS
Symptomatic	6 (12.0%)	2 (8.0%)	8 (10.7%)	NS
ER/Hospital Visit for COVID related symptoms	0 (0.0%)	2 (8.0%)	2 (2.7%)	NC
If asymptomatic, patient became symptomatic within 14 days of testing	1 (2.0%)	4 (16.7%)	5 (6.8%)	NC
Underwent planned procedure				
Yes	49 (98%)	15 (60%)	64 (85.3%)	< 0.001
No	1 (2.0%)	10 (40.0%)	11 (14.7%)	

**Table 2:** Effects on planned procedure on the subset of cases who underwent their planned procedure and respective matched controls. Cases: positive COVID-19 screening, Control: negative COVID-19 screening

	Cases N (%)	Controls N (%)	p-value
N	15 (33.3%)	30 (66.7%)	
Delay related to positive COVID-19 test			
Yes	11 (73.3%)	0 (0%)	<0.001
No	4 (26.7%)	30 (100%)	
Delay (days)			
Mean (SD)	18 (16.7)	0 (0)	
Range	0 – 49	0–0	
